# Smoking uptake and prevalence in Ghana

**DOI:** 10.1136/tc.2009.030635

**Published:** 2009-07-05

**Authors:** E Owusu-Dabo, S Lewis, A McNeill, A Gilmore, J Britton

**Affiliations:** 1 UK Centre for Tobacco Control Studies, Division of Epidemiology and Public Health, University of Nottingham, Clinical Sciences Building, City Hospital, Nottingham, UK ^2^Department of Community Health, School of Medical Sciences, College of Health Sciences, Kwame Nkrumah University of Science and Technology, Kumasi, Ghana; 2 School for Health, University of Bath, Bath and London School of Hygiene and Tropical Medicine, University of London, UK

## Abstract

**Background:**

Developing countries are at high risk of epidemic increases in tobacco smoking, but the extent of this problem is not clearly defined because few collect detailed smoking data. We have surveyed tobacco smoking in the Ashanti region of Ghana, a rapidly developing African country with a long-established tobacco industry.

**Methods:**

We took a random sample of 30 regional census enumeration areas, each comprising about 100 households, and a systematic sample of 20 households from each. These were visited, a complete listing of residents obtained and questionnaire interviews on current and past smoking, age at smoking uptake, sources of cigarettes and other variables carried out in all consenting residents aged 14 or over.

**Results:**

Of 7096 eligible individuals resident in the sampled households, 6258 (88%; median age 31 (range 14–105) years; 64% female) participated. The prevalence of self-reported current smoking (weighted for gender differences in response) was 3.8% (males 8.9%, females 0.3%) and of ever smoking 9.7% (males 22.0%, females 1.2%). Smoking was more common in older people, those of Traditionalist belief, those of low educational level, the unemployed and the less affluent. Smokers were more likely to drink alcohol and to have friends who smoke. About 10% of cigarettes were smuggled brands. About a third of smokers were highly or very highly dependent.

**Conclusions:**

Despite rapid economic growth and a sustained tobacco industry presence, smoking prevalence in Ghana is low, particularly among younger people. This suggests that progression of an epidemic increase in smoking has to date been avoided.

Smoking is the largest avoidable cause of death and major non-communicable disease worldwide.^1^ ^2^ Although historically prevalent in economically developed countries and rare in the developing world, this pattern of smoking is now beginning to reverse as smoking prevalence rises in the developing world, particularly among men.^3^ Consequently, approximately 70% of the 10 million deaths from tobacco-related causes expected by 2030 will occur in developing countries,^4^ where epidemic increases in smoking prevalence will inflict major public health impacts throughout the 21st century.^5^ ^6^ Smoking surveillance and prevention should therefore be high priorities in these countries,^1^ but although many have committed to implement the World Health Organization Framework Convention on Tobacco Control (FCTC),^7^ the tobacco control resources available to do so are typically very limited. Reliable data on country prevalence and trends in prevalence in the developing world are few.^8^

Ghana is a country in sub-Saharan Africa with a population of about 22 million, a gross national income (in 2005) of $450 per capita, has had a stable democracy since 1992 and recent economic growth of around 6% per year.^9^ British American Tobacco has had a manufacturing presence in Ghana for most of the past 50 years.^10^ Ghana is therefore likely to be at a relatively high risk of involvement in the tobacco epidemic, but detailed information on smoking in the general adult population is not available. This study was carried out in the most populous region of Ghana to investigate the extent of tobacco use in Ghana, measuring current and ever smoking, age and year of uptake of smoking, the demographic characteristics of smokers, the main risk factors for smoking and main sources of tobacco, smoking cessation patterns and other variables, to characterise and describe the extent to which tobacco smoking has become prevalent.

## Materials and methods

### Study site

Ghana is situated a few degrees north of the Equator on the West African Gulf of Guinea, has a total area of 238 540 km^2^ and a current population of about 22 million. The Ashanti region, where the study was carried out, is located in central area of Ghana and, with a population of over 4.4 million people in 2006,^11^ is the most heavily populated of the 10 regions of the country. The region’s demographic characteristics are comparable with national data,^12^ suggesting that it is broadly representative of the country.

### Sample design

We used two-stage cluster randomised sampling to recruit a representative sample of Ashanti Region residents aged 14 years and above. Our initial sampling frame was the list of Ashanti Enumeration Areas (EA) from the 2000 Ghana population and Housing Census; EAs are population counting units, each typically comprising about 100 households. We stratified the list of EAs into rural and urban, and took a random sample of 30 (15 urban and 15 rural) EAs for study.

Fieldworkers from the Ghana Statistical Service of the Ashanti Region were then engaged to visit each sampled EA, and to identify a 20% systematic sample of houses in each EA by walking a serpentine route through the EA and making a chalk mark on the wall of every fifth house. These houses were then visited by trained fieldworkers who explained the study and obtained informed consent (with approval given by way of signature or thumbprint as appropriate) before conducting a face-to-face interview separately of all individuals aged 14 and over using structured questionnaires. Up to three repeat visits were made to capture data for individuals who were not present at the first visit. We excluded individuals living in institutions (such as hospitals, prisons and hotels) and foreign nationals. The survey was completed between December 2007 and May 2008.

### Data collection and study variables

The smoking questions were based on those used in the UK General Household Survey^13^ and the International Tobacco Control survey,^14^ supplemented by questions on local issues relevant to smoking. The questionnaires were translated and back-translated from English into local Asante Twi language and pilot tested in a sample of 20 households outside the study area. Questions covered demographic and socioeconomic variables, cigarette smoking and, for anyone who has smoked, brand, price paid and usual place of purchase, age at onset of smoking, duration of regular (at least daily) smoking, current use of tobacco products other than cigarettes, the number of close friends who smoke and other variables. A current smoker was defined as someone who reported that they had smoked at least 100 cigarettes over his or her lifetime and smokes nowadays; an ex-smoker one who had smoked at least 100 cigarettes but reported never smoking a cigarette, cigar or pipe now. We used questions on ownership of consumer goods to categorise socioeconomic status. Dependence on smoking and tobacco use were measured using the modified Fagerstrom score,^15^ from a set of six questions assessing smoking addiction on a 10-point scale. Dependence was classified from a maximum score of 10 into very low (0–2), low (3–4), medium (5), high (6–7) and very high (8–10). We also asked a range of questions covering smoking policy at respondent’s place of work, support for smoking regulations in indoor public areas, knowledge of health effects and diseases caused by smoking, beliefs about the dangers of different tobacco products and other factors. These data will be reported separately.

### Sample size and power

Ashanti has a 51% urban and 49% rural population. Sampling urban and rural EAs in a 1:1 ratio, and anticipating (from estimates based on local knowledge) that smoking prevalence would be of the order of 5% to 10%, and a design effect (that is, a multiplication factor for sample size to allow for clustering within household) of up to 2, a total sample of 6000 participants was estimated to allow us to estimate the prevalence of smoking in the urban area to within 1% and in the rural area (where prevalence was expected to be slightly lower) to within 2%. The sample size also provides over 95% power to detect a twofold or greater difference in prevalence between urban and rural areas, or between exposed and unexposed individuals, for any risk factor occurring in at least 20% of the population, taking an α of 5%.

### Ethics approvals

Approval for the study was granted by the committee of human research and ethics of the School of Medical Sciences of the Kwame Nkrumah University of Science and Technology, Kumasi; by the ethics review board of the Ghana Health Service in Accra; and the local ethics committee of the University of Nottingham, UK.

### Data analysis

Data were analysed using Stata SE version 10, and using the survey commands to allow for the sampling design, including the stratification by locality, and the clustering by EA and household, and to weight for gender differences in participation in relation to the ascertained study sample, and also in relation to the gender distribution reported in national survey data. Proportions and 95% confidence intervals were obtained as estimates of prevalence. Multivariate analyses of predictors of smoking status were conducted using logistic regression, adjusting for age, gender and urban/rural locality type where appropriate.

## Results

### Characteristics of study participants

Of the 7096 individuals (2900 males, 4196 females) ascertained to be members of the sampled households and thus eligible for the study, 6258 (88%; 78% of men and 95% of women) participated. Of these, 2274 (36.3%) were male and 3984 (63.7%) were female. The median age of participants was 31 (range 14–105). In both urban and rural areas, the median number of household members among study participants was 3 (range 1–18). Table 1 summarises the sociodemographic characteristics of the study participants and illustrates the relatively high proportions of participants who were female, from younger adult age groups, from the Akan ethnic group, who were self employed, had attained secondary level of education and who described themselves as of the Christian faith.

**Table 1:** Demographic distribution of study population and prevalence and determinants of current smoking

Characteristic	Number (%)	Males	Smokers			Female		Smokers	Adjusted odds ratio (95% CI)		p Value
		No (%)	No (%)			No (%)		No (%)			
**Locality type**											
**Total**	**6258 (100)**	**2274 (36.33)**		**202 (3.23)**	**3984 (63.66)**		**11 ( 0.18)**			1	
Urban	3161 (50.5)	1134 (35.87)		106 (3.4)	2027 (64.13)		7 (0.22)			0.90 (0.64 to 1.27)	**0.54**†
Rural	3097 (49.5)	1140 (36.81)		96 (3.1)	1957 (63.19)		4 (0.13)				
**Age (years)**											
14–19	1144 (18.3)	478 (41.78)		10 (0.87)	666 (58.22)		2 (0.17)			1	
20–29	1686 (26.9)	595 (35.29)		39 (2.31)	1091 (64.71)		2 (0.12)			2.44 (1.19 to 4.98)	
30–39	1277 (20.4)	450 (35.24)		51 (4.00)	827 (64.76)		3 (0.23)			4.32 (2.10 to 8.90)	
40–49	810 (12.9)	277 (34.20)		35 (4.32)	533 (65.80)		1 (0.12)			4.59 (2.67 to 7.87)	<0.001*
50–59	554 (8.9)	193 (34.84)		35 (6.32)	361 (65.16)		0 (0.00)			6.69 (3.41 to 12.38)	
60–69	328 (5.2)	121 (36.89		19 (5.79)	207 (63.11)		1 (0.30)			6.36 (3.26 to 12.38)	
⩾70	459 (7.3)	160 (34.86)		13 (2.83)	299 (65.14)		2 (0.44)			3.26 (1.43 to 7.42)	
**Religion**											
Christian	5699 (91.5)	2003 (35.15)		152 (2.67)	3696 (64.85)		9 (0.16)			1	
Muslim	424 (6.8)	165 (38.91)		15 (3.54)	259 (61.08)		2 (0.47)			1.37 (0.84 to 2.22)	**<0.001**†
Traditionalist	82 (1.3)	66 (80.49)		29 (35.33)	16 (19.51)		0 (0.00)			7.50 (4.43 to 12.69)	
Other	53 (0.8)	40 (75.47)		6 (11.3)	13 (24.53)		0 (0.00)			1.80 (0.67 to 4.84)	
**Education**											
Illiterate	1004 (16)	210 (20.19)		25 (2.49)	794 (79.08)		3 (0.30)			1	
Primary	765 (12.2)	208 (27.19)		35 (4.58)	557 (72.81)		1 (0.13)			1.49 (0.81 to 2.73)	**0.03**†
Secondary	4206 (67.2)	1667 (39.64)		129 (3.07)	2539 (60.37)		7 (0.17)			0.74 (0.38 to 1.43)	
Tertiary	283 (4.5)	189 (66.78)		13 (4.59)	94 (33.22)		0 (0.00)			0.53 (0.20 to 1.40)	
**Ownership of goods**											
None	1462 (23.3)	363 (24.83)		29 (1.98)	1099 (75.17)		6 (0.41)			1	
Radio	1262 (20.2)	513 (40.65)		58 (4.59)	749 (59.02)		1 (0.07)			1.18 (0.75 to 1.85)	
Telephone	1513 (24.2)	598 (39.52)		34 (2.25)	1096 (72.44)		0 (0.00)			0.53 (0.29 to 0.97)	**0.07**†
TV	1694 (27.1)	654 (38.61)		71 (4.19)	859 (50.71)		4 (0.24)			1.09 (0.59 to 2.03)	
Car	327 (5.2)	146 (44.65)		10 (3.06)	181(55.35)		0 (0.24)			0.61 (0.27 to 1.39)	
**Occupation**											
Unemployed	1231 (19.7)	330 (26.81)		39 (3.13)	901 (73.19)		5 (0.41)			1	
Self-employed	2403 (38.4)	679 (28.26)		59 (2.46)	1724 (71.74)		4 (0.17)			0.53 (0.36 to 0.79)	
Student	852 (13.6)	418 (49.06)		6 (0.70)	434 (50.93)		1 (0.12)			0.19 (0.08 to 0.41)	
Administrative staff	99 (1.6)	59 (59.60)		7 (7.07)	40 (40.40)		0 (0.00)			0.71 (0.27 to 1.83)	**0.003**†
Urban skilled worker	652 (10.4)	426 (65.34)		41 (6.23)	226 (34.67)		0 (0.00)			0.66 (0.41 to 1.06)	
Rural worker (farmer)	1021 (16.3)	362 (35.46)		50 (4.89)	659 (64.54)		1 (0.09)			0.90 (0.52 to 1.55)	
**Ethnicity**											
Akan	5423 (86.7)	1969 (36.31)		171 (3.15)	3454 (63.69)		8 (0.14)			1	
Ewe	59 (0.9)	20 (33.40)		3 (5.08)	39 (66.10)		1 (1.69)			2.54 (0.73 to 8.90)	**0.33**†
Dagbani	43 (0.7)	17 (39.53)		3 (6.98)	26 (60.47)		0 (0.00)			1.52 (0.56 to 4.12)	
Others	733 (11.7)	268 (36.56)		25 (3.41)	465 (63.44)		2 (0.27)			1.17 (0.78 to 1.77)	
**Alcohol use**											
Yes	2165 (34.60)	1132 (52.29)		177 (8.18)	1033 (47.71)		9 (0.42)			1	**<0.001**†
No	4093 (65.40)	1142 (27.90)		25 (0.61)	2951 (72.10)		2 (0.05)			0.13 (0.08 to 0.22)	
**Friends who smoke**											
None	5100 (81.50)	1516 (29.73)		29 (0.57)	3584 (70.27)		3 (0.01)			1	
1–3	708 (11.31)	415 (58.62)		7 (0.99)	293 (41.38)		2 (0.28)			11.23 (6.57 to 19.21)	**<0.001**†
>3	450 (7.19)	343 (76.22)		96 (21.33)	107 (23.78)		6 (1.33)			21.72 (13.36 to 35.31)	
**Exercise/week**											
Never	3109 (49.68)	794 (25.54)		104 (3.35)	2315 (74.46)		10 (0.32)			1	
Once	711 (11.36)	283 (39.80)		19 (2.67)	428 (60.20)		0 (0.00)			0.49 (0.26 to 0.94)	
More than once	1115 (17.82)	510 (45.93)		21 (1.88)	605 (54.26)		0 (0.00)			0.30 (0.19 to 0.47)	**<0.001**†
Everyday	1323 (21.14)	687 (51.93)		58 (4.38)	636 (48.07)		1 (0.08)			0.65 (0.45 to 0.96)	

*p value for trend.

†Wald p value adjusted for age, gender and urban or rural locality type.

### Prevalence and amount of smoking

Current smoking was reported by 202 (8.9%; 95% confidence interval 7.3% to 10.5%) males and 11 (0.3%; 0.1% to 0.4%) females. The unadjusted overall prevalence of smoking was 3.4% (3.0% to 3.9%), and the prevalence adjusted for male under-response 3.8% (3.1% to 4.4%). Adjustment for male under-representation in the study sample, by population-based weighting using national survey data,^12^ increased the estimate of current smoking prevalence by about 0.9 percentage points, to 4.3% (3.6% to 5.0%). The unadjusted prevalence of ever smoking was 8.7% (8.1% to 9.5%, comprising 22.0% (19.3% to 24.8%) of males and 1.2% (0.7% to 1.6%) of females), and the prevalence adjusted for male under-response was 9.7% (8.4% to 10.9%). There were 334 ex-smokers, the numbers of which were distributed evenly across all ages over 20 years, with age-specific proportions that increased from 1.8% in the 14–19 age group to 12.6 in the over 70 age group (table 2).

**Table 2:** Numbers of current smokers, ex-smokers and ever smokers, and proportion of ever smokers who are ex-smokers, by age

Age (years)	No	Current smokers	Ex-smokers	Ever smokers	Ex-smokers as % of total smokers
		No (%)	No (%)	No (%)	
14–19	1144	12 (1.0)	21 (1.8)	33 (2.9)	63.6
20–29	1686	41 (2.4)	56 (3.3)	97 (5.8)	57.7
30–39	1277	54 (4.2)	54 (4.2)	108 (8.5)	50.0
40–49	810	36 (4.4)	53 (6.5)	89 (11.0)	59.6
50–59	554	35 (6.3)	53 (9.6)	88 (15.9)	60.2
60–69	328	20 (6.1)	39 (11.9)	59 (18.0)	66.1
⩾70	459	15 (3.3)	58 (12.6)	73 (15.9)	79.5

The median number of cigarettes smoked per day by male and female current smokers was respectively 6 (interquartile range 1–40) and 5 (4–10) on weekdays, and 19 (2–70) and 11 (8–20) at the weekend.

### Determinants of smoking

Table 1 also details the associations between current smoking and various sociodemographic and other characteristics. Smoking prevalence was similar in urban and rural areas and across ethnic groups, and was higher in men, in older age groups (other than in those aged over 70 years), among those of Traditionalist faith, among the unemployed and those with no or only primary education. Smoking was slightly but not significantly more common in those who owned a radio or television, and lower in those who owned a telephone or car. Smokers were more likely to drink alcohol, to have friends who smoke and significantly less likely to take regular exercise.

Age at uptake of smoking, sources of tobacco products, level of addiction and other characteristics of current smokers are shown in table 3. Just under half of all current smokers started smoking before the age of 20 and 87% by the age of 30. Cigarettes tended to be purchased from drinking spots or pubs rather than shops, and the majority of brands purchased were those marketed in Ghana by BAT Ghana Ltd. Brands that are not marketed in Ghana but are available in neighbouring countries (*Bond* and *Gold Seal* from Togo, *Craven* from Côte d’Ivoire) accounted for about 10% of purchases. Over 40% of smokers spent more than 1 Ghanaian cedi (GHC; 1 GHC being approximately equivalent to $US 1) on cigarettes per day. Over a third of smokers had high to very high dependence on cigarettes. Approximately a quarter of current smokers had used smokeless tobacco in the past 6 months.

**Table 3:** Smoking characteristics of current smokers in study

Characteristic	No (%)
Age of uptake of smoking	
0–9	2 (0.93)
10–19	102 (47.89)
20–29	82 (38.50)
30–39	20 (9.39)
40–49	7 (3.29)
Sources of cigarettes	
Shops	58 (27.23)
Pubs and drinking spots	144 (67.61)
Roadside	11 (5.16)
Primary brand (manufacturer) of cigarettes used	
*State Express 555* (BAT)	27 (12.68)
*Bond* (smuggled, originating in Togo)	12 (5.63)
*Diplomat* (BAT)	8 (3.76)
*Gold Seal* (smuggled, originating in Togo)	10 (4.69)
*King Size* (BAT)	46 (21.60)
*London Brown* (BAT)	91 (42.72)
*London White* (BAT)	12 (5.63)
*Pall Mall* (BAT)	6 (2.82)
*Craven* (smuggled from Côte d’Ivoire)	1 (0.50)
Form of tobacco used*	
Manufactured cigarettes	213 (100)
Pipes	6 (2.82)
Cigar	17 (7.98)
Smokeless (moist snuff, nasal snuff, chew)	54 (25.35)
Amount spent on cigarette/day (in Ghanaian cedis)	
Less than 0.5	76 (35.98)
0.5–1	50 (23.36)
1.1–5	62 (28.97)
>5	25 (11.68)
Levels of addiction	
Very low dependence	25 (11.27)
Low dependence	80 (37.74)
Medium dependence	27 (12.74)
High dependence	56 (26.42)
Very high dependence	24 (11.32)

*Multiple responses (in addition to cigarettes could respond to any).

**Figure 1:**
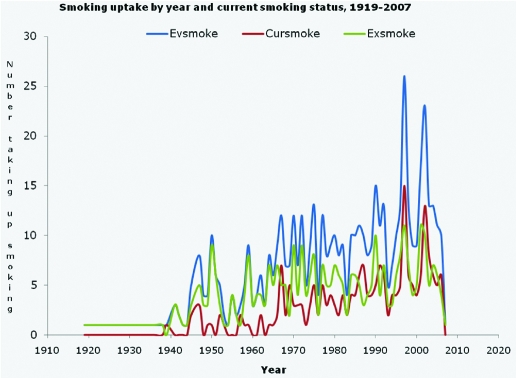
Year of uptake of smoking in ever smokers, current smokers and ex-smokers.

The number of smokers in the study sample taking up smoking each year, grouped by smoking status into ever, current and ex-smokers is shown in figure 1. The plot demonstrates that within this predominantly young study population, with the exception of two peak years since 2000, the numbers taking up smoking rose only slowly over time. The proportion of ever smokers who were ex-smokers was broadly similar across most of the age range of respondents (table 2).

## Discussion

This is the first study to provide detailed data on the prevalence and determinants of smoking in Ghana. Our finding of higher smoking prevalence among men is consistent with previous studies in Ghana^8^ ^16^ ^17^ ^18^ ^19^ and elsewhere in Africa,^8^ ^20^ ^21^ ^22^ ^23^ while our overall adjusted estimates of current smoking prevalence, at 3.8%, and of ever smoking at 9.7%, indicate that the prevalence of smoking in Ghana is one of the lowest in Africa.^8^ Our data on self-reported age at uptake of smoking, combined with the lower rates of current smoking among younger than older age groups and the high proportion of ex-smokers in all age groups, suggest that population uptake has increased only slowly during the past 50 years or so, and therefore that Ghana has thus far avoided the marked increase in smoking prevalence that often accompanies economic development.

We limited our study to the Ashanti region of the country for logistic reasons, but chose this region because it is the most heavily populated of the 10 regions of Ghana, and is generally representative of the national population.^12^ Participation was high, at 88%, though higher in females than males, and we weighted our prevalence estimates to allow for this non-response. Nevertheless, the male-to-female ratio of our sampled population at 59:41 differed from the national ratio of 53:47.^19^ The reasons for the male under-representation is not clear; we attempted to ascertain the names of all long-term residents (defined as any person who had lived at the address for at least one year) in each household, and one possibility is that a substantial proportion of men had left or were otherwise long-term absentees from the family home. We have no further data to assess whether the smoking behaviour of the men in our sample population is representative of men in Ashanti, or in the wider Ghanaian population. However, allowing for male under-representation relative to the national population increased our prevalence estimate by only 0.9 percentage points. We attempted to minimise the possibility that responses would be affected by social pressures against smoking, particularly on women and young people, by interviewing respondents individually, and as privately as possible. However, it is possible that such pressures have resulted in a degree of underestimation of true prevalence in those groups.

Previous studies of smoking in Ghana are few and have involved either very small and unrepresentative samples,^24^ ^25^ ^26^ have been limited to young people,^27^ or in the case of the one national survey to date, based on responses to only three questions.^19^ However the findings of these studies are all broadly consistent with ours, indicating that the prevalence of smoking in Ghana is indeed low in absolute terms, and in relation to other countries in sub-Saharan Africa.^8^ The low prevalence of smoking among women is a typical finding in African countries,^8^ ^23^ ^28^ and has been attributed to limitation of opportunities to smoke by low levels of economic independence among women,^28^ ^29^ and sociocultural contexts within which smoking among women is often considered to be immoral.^30^ ^31^ Our observation that current smokers are likely to have friends who smoke, tend to use alcohol and to be less physically active, is also consistent with other reports.^32^ ^33^ That cigarette consumption almost doubled over the weekend perhaps also reflects the influence of social pressures or incentives to smoke. Our finding that smoking is more prevalent among the less well educated, less economically active and relatively less affluent members of the population contrasts with the typical pattern of the early stages of uptake of smoking in most countries, including many developing countries,^34^ where smoking of manufactured cigarettes often occurs first among those in paid employment.^16^ Our findings demonstrate that smoking imposes a substantial economic cost on those who smoke, since most smokers spend more than one Ghanaian cedi on smoking per day; this represents about a third of the likely wage of the typical smoker in our study.^35^ That approximately 10% of all cigarettes purchased by our study population were brands that are not marketed in Ghana affirms the longstanding problem of smuggling in Ghana, particularly from Togo.^8^ ^36^

Ghana has enjoyed consistent economic growth over recent years, and from the time that Ghana first became independent in 1957 until as recently as 2006, British American Tobacco has had an active manufacturing facility in the country. Given this relative affluence and a local source of manufactured cigarettes from an early stage, Ghana might therefore be expected to be at a relatively advanced stage of the tobacco epidemic in relation to other developing countries, and to have a relatively high prevalence of smoking. The picture emerging from our data is however more complex, perhaps reflecting the inability of the traditional model of the tobacco epidemic, based on trends observed in the West^37^ to adequately explain patterns elsewhere. Thus, although the low rates of smoking and gender disparity suggest Ghana may be at an early stage of the epidemic, most aspects of our data (the significant proportion of ex-smokers, lower rates of smoking in younger than older age groups and lower rates among the more educated), are all consistent with Ghana being at a relatively late stage, and therefore perhaps avoiding the expected high prevalence of smoking. Results consistent with this interpretation, reporting a fall in smoking prevalence among civil servants in Accra, have been reported elsewhere.^38^

Cultural factors may have had a strong influence on the pattern of smoking in Ghana. Ghana is a religious country with substantial Christian (70%) and Muslim (17%) populations,^19^ among whom smoking was relatively uncommon in our study, in contrast with those of Traditionalist (a belief in the existence of lesser gods, with adherence to traditional Ghanaian customs) faith. Whether these associations are causal, and if so how, is not clear from our data. We have also reported elsewhere^10^ that Ghana’s low smoking prevalence and tobacco use may also have been attributable to the imposition of partial government ownership of the main tobacco company in Ghana at the early stage of epidemic growth in smoking prevalence in the 1970s and 1980s, and to a lack of foreign exchange to fund tobacco leaf importations in addition to implementation of a ban on tobacco advertising in 1982.^39^ ^40^ ^41^ In reality, Ghana’s experience of low tobacco use and smoking prevalence to date probably reflects a broad mix of cultural and political influences, and does not necessarily imply that a more marked increase in smoking prevalence will not occur in the future. It remains to be seen whether Ghana’s experience will be replicated in other developing countries in Africa and elsewhere in the world.

**What this paper adds**

- This study provides the first comprehensive assessment of the prevalence and determinants of smoking in a Ghanaian population.
- It shows that despite economic growth and an active tobacco industry presence in Ghana, smoking prevalence remains very low.
- The findings suggest that Ghana’s smoking prevalence may have already peaked and declined and that a major smoking epidemic is not inevitable.
- Possible reasons include cultural factors, partial state ownership of the tobacco industry, timely cigarette shortages and an early tobacco advertising ban.

## References

[1] Ezzati M Henley SJ Lopez AD Role of smoking in global and regional cancer epidemiology: current patterns and data needs. Int J Cancer 2005;116:963–71 15880414 10.1002/ijc.21100

[2] 2004 Surgeon General’s report links more cancers to smoking. Ca-A Cancer J Clin 2004;54:243–4

[3] Mackay J Eriksen M The tobacco atlas. Geneva: World Health Organization, 2002

[4] Emmanuel Guindon G Bosclair D Past, current and future trends in tobacco use. The International Back for Reconstruction and Development/World Bank, 2003

[5] Mufunda J Chatora R Ndambakuwa Y Prevalence of noncommunicable diseases in Zimbabwe: results from analysis of data from the National Central Registry and Urban Survey 2. Ethn Dis 2006;16:718–22 16937610

[6] Danaei G Vander Hoorn S Lopez AD Causes of cancer in the world: comparative risk assessment of nine behavioural and environmental risk factors. Lancet 2005;366:1784–93 16298215 10.1016/S0140-6736(05)67725-2

[7] World Health Organization WHO Framework Convention on Tobacco Control. Geneva: WHO, 2003

[8] Pampel F Tobacco use in sub-Sahara Africa: estimates from the demographic health surveys. Soc Sci Med 2008;66:1772–83 18249479 10.1016/j.socscimed.2007.12.003 PMC2679748

[9] Ministry of Finance Economic Planning G Budget statement of Ghana, 2008; a brighter future. http://ghanagovgh/files/budget%202008pdf, 2008 (accessed 16 October 2008)

[10] Owusu-Dabo E Lewis S McNeill A Smoking in Ghana: a review of tobacco industry activity. Tob Control 2009;18:206–11 19359263 10.1136/tc.2009.030601 PMC2679188

[11] Regional Health Directorate GHS Annual Report Ashanti Region. 2007

[12] Ghana Statistical Service 2000 Population and housing census. Summary report of final results. Accra, Ghana, 2002

[13] Office for National Statistics General household survey, 2005: smoking and drinking among adults. wwwstatisticsgovuk/ghs, 2006 (accessed 12 March 2007). Available from www.statistics.gov.uk/ghs

[14] Fong G Borland R Cummings M International Tobacco Control (ITC) policy survey. wwwitcproject org (accessed 12 June 2007). Available from www.itcproject.org 10.1136/tc.2005.015438 PMC2593053 16754944

[15] Heatherton TF Kozlowski LT Frecker RC The Fagerstrom test for nicotine dependence: a revision of the Fagerstrom Tolerance Questionnaire. Br J Addict 1991;86:1119–27 1932883 10.1111/j.1360-0443.1991.tb01879.x

[16] Blakely T Hales S Kieft C The global distribution of risk factors by poverty level 74. Bull World Health Organ 2005;83:118–26 15744404 PMC2623808

[17] Amonoolartson R Pappoe ME Prevalence of smoking in secondary-schools in the Greater Accra Region of Ghana 6. Soc Sci Med 1992;34:1291–3 1641687 10.1016/0277-9536(92)90321-g

[18] Amoah AG Owusu SK Adjei S Diabetes in Ghana: a community based prevalence study in Greater Accra 17. Diabetes Res Clin Pract 2002;5:197–205 11947967 10.1016/s0168-8227(01)00374-6

[19] Ghana 2003: results from the demographic and health survey. Studies in Family Planning 2005;36:158–62 15991656 10.1111/j.1728-4465.2005.00056.x

[20] Kenya 2003: results from the demographic and health survey. Studies in Family Planning 2005;36:163–7 15991657 10.1111/j.1728-4465.2005.00057.x

[21] Brook JS Morojele NK Brook DW Predictors of cigarette use among South African adolescents. Int J Behav Med 2005;12:207–17 16262539 10.1207/s15327558ijbm1204_1 PMC1343497

[22] Muula AS Prevalence and determinants of cigarette smoking among adolescents in Blantyre City, Malawi. Tanzan Health Res Bull 2007;9:48–51 17547101 10.4314/thrb.v9i1.14292

[23] Rudatsikira E Abdo A Muula AS Prevalence and determinants of adolescent tobacco smoking in Addis Ababa, Ethiopia. BMC Public Health 2007;7:176 17651482 10.1186/1471-2458-7-176 PMC1940247

[24] Corti A, INFOTAB Mbabane Conference on 26–28th April 1982. http://batlibrary ucsf edu/tid/buo21a99, 1982 (accessed 24 July 2007)

[25] Yach D Warren CW Silva VLD Tobacco use among youth: a cross country comparison 14. Tob Control 2002;11:252–70 12198280 10.1136/tc.11.3.252 PMC1759013

[26] Marketing News. http://batlibraryucsfedu/tid/itg64a99, 1973 (accessed 23 July 2007)

[27] Warren CW Riley L Asma S Tobacco use by youth: a surveillance report from the Global Youth Tobacco Survey project 186. Bull World Health Organ 2000;78:868–76 10994259 PMC2560802

[28] Kaplan M Carriker L Waldron I Gender differences in tobacco use in Kenya. Soc Sci Med 1990;30:305–10 2309128 10.1016/0277-9536(90)90186-v

[29] Waldron I Bratelli G Carriker L Gender differences in tobacco use in Africa, Asia, the Pacific, and Latin-America. Soc Sci Med 1988;27:1269–75 3206258 10.1016/0277-9536(88)90357-7

[30] Barraclough S Women and tobacco in Indonesia. Tob Control 1999;8:327–32 10599579 10.1136/tc.8.3.327 PMC1763945

[31] Mackay J Amos A Women and tobacco. Respirology 2003;8:123–30 12753525 10.1046/j.1440-1843.2003.00464.x

[32] Nagaya T Yoshida H Takahashi H Cigarette smoking weakens exercise habits in healthy men. Nicotine Tob Res 2007;9:1027–32 17943618 10.1080/14622200701591575

[33] Rodrigues ES Cheik NC Mayer AF Level of physical activity and smoking in undergraduate students. Revista de Saude Publica 2008;42:672–8 18709243 10.1590/s0034-89102008000400013

[34] Bobak M Jha P Nguyen S Poverty and smoking. Tobacco control in developing countries. Jha P Chaloupka F, eds. Oxford: Oxford University Press, 2000:41–61

[35] National Development Planning Commission Growth and poverty reduction strategy (GPRS II) 2006–2009. http://siteresourcesworldbankorg/INTPRS1/Resources/Ghana_PRSP(Nov-2005)pdf, 2005 (accessed 1 July 2008)

[36] Koomson J Smuggling in Ghana cost government over US$ 3.5 million annually. Ghanaian Chronicle 2006 Aug 26.

[37] Lopez AD Collishaw NE Piha T A descriptive model of cigarette epidemic in developed countries. Tob Control 1994;3:242–7

[38] Addo J Smeeth L Leon DA Smoking patterns in Ghanaian civil servants: changes over three decades. Int J Eviron Res Public Health 2009;6:200–8 10.3390/ijerph6010200 PMC2672323 19440277

[39] Selby R SE555 Lights and TV Advertising. http://batlibraryucsfedu/tid/sfx11a99, 1994 (accessed 25 July 2007)

[40] Offer for Sale-PTC SHARES. http://batlibraryucsfedu/tid/qfg96a99, 1976 (accessed 24 July 2007)

[41] Bluett I Ghana. http://batlibraryucsfedu/tid/vby97a99, 1976 (accessed 24 July 2007)

